# Supplementary material for: Endothelial Cells Tissue-Specific Origins Affects Their Responsiveness to TGF-β2 during Endothelial-to-Mesenchymal Transition
Source: Int J Mol Sci. 2019 Jan 22;20(3):458. doi: 10.3390/ijms20030458 (PMC6387078; doi:10.3390/ijms20030458)
Supplement: Supplementary file 1 [file ijms-20-00458-s001.pdf]

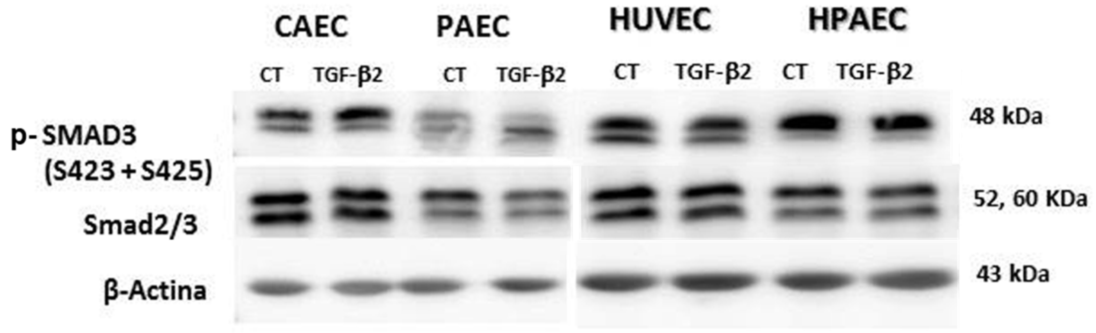

Figure S1. Smad-3 expression analysis of endothelial cells treated or not with TGF-β2. Western blotting for phospho-Smad 3 (S423/S425) and total Smad 2/3. β-actin was used as endogenous control (representative image of one replicate of each sample).

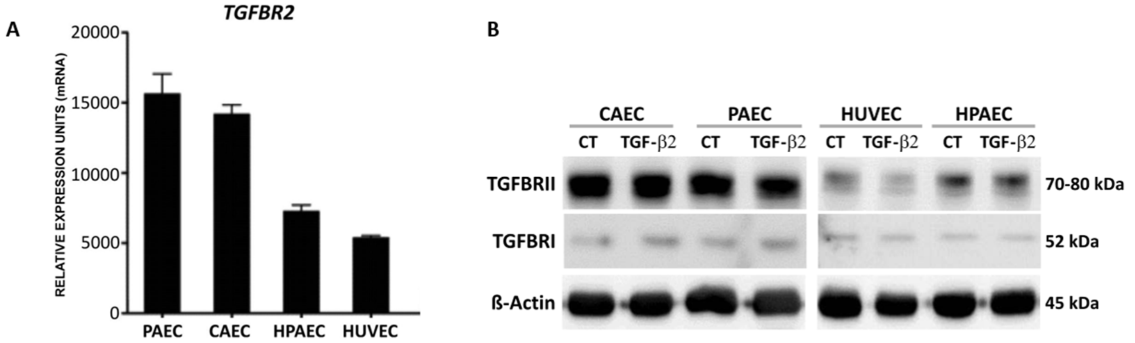

Figure S2. TGF-β receptor I (TGFBRI) and II (TGFBRII) expression analyzes of endothelial cells treated or not with TGF-β2. **(A)** TGFBRII gene expression analysis by qPCR on PAEC, CAEC, HPAEC, and HUVEC. The bars represent the relative expression unit values. **(B)** TGFβ western blotting receptors in EC treated or not with TGF-β2. β-actin was used as endogenous control (representative image of one replicate of each sample).
